# Reply: Regarding Racial Disparities in Door-to Clinician Time for Cardiac Chest Pain

**DOI:** 10.5811/westjem.63979

**Published:** 2026-07-16

**Authors:** Emad Awad

**Affiliations:** University of Utah Spencer Fox Eccles School of Medicine, Department of Emergency Medicine, Salt Lake City, Utah

To the Editor:

We thank the letter writers for their thoughtful engagement with our study.^1^ Their comments are well taken, align with several limitations we acknowledged, and highlight important directions for future research.

We agree that language discordance may contribute to delays in care through mechanisms such as interpreter access, which are distinct from individual clinician bias. Our model did not capture language variables, and we support prospective collection of preferred language and interpreter use in future studies. We also agree that Emergency Severity Index (ESI) assignment may function not only as a confounder, but also as a potential mediator if triage decisions are themselves influenced by race or ethnicity. In our analysis, we adjusted for triage acuity to estimate associations independent of ESI level. However, mediation analysis in larger cohorts may better clarify these pathways.

As noted in our article, the relatively small sample of Black patients (n = 213) limits the precision of subgroup estimates. The confidence interval (0.94–1.30) remains compatible with clinically meaningful disparities, and the absence of statistical significance should not be interpreted as evidence of equity.

The letter writers also appropriately note that our 2019–2025 study period spanned the COVID-19 pandemic, during which emergency department operations changed substantially. As with most observational studies, however, complete temporal control is difficult without compromising model stability, particularly in smaller subgroups. Stratifying by period or introducing multiple interaction terms would have reduced effective sample size and increased the risk of unstable estimates. As George Box observed, “All models are wrong, but some are useful.”^2^ Our aim was to provide a useful real-world estimate of disparities in door-to-clinician time, while recognizing that unmeasured temporal factors may remain. Larger multisite studies will be better positioned to evaluate whether disparities vary across operational periods.

Several of the issues raised by the letter writers, including language discordance and sample size limitations, were acknowledged in our discussion. We appreciate their further elaboration on these points and their emphasis on future work.

We value this dialogue and join the letter writers in calling for larger, multisite studies with richer clinical, language, and operational variables to advance equitable emergency cardiac care.

## References

[1] Awad E, Raju S, Alsayyed H (2026). Racial disparities in door-to-clinician time for cardiac chest pain in the emergency department. West J Emerg Med.

[2] Box GEP, Draper NR (1987). Empirical Model-Building and Response Surfaces.

